# Supplementary material for: RNA G-quadruplexes function as a tunable switch of FUS phase separation
Source: Nucleic Acids Res. 2026 Jun 27;54(12):gkag594. doi: 10.1093/nar/gkag594 (PMC13309791; doi:10.1093/nar/gkag594)
Supplement: gkag594_Supplemental_File [file gkag594_supplemental_file.pdf]

SUPPLEMENTARY MATERIAL RELATED TO

RNA G-Quadruplexes Function as a Tunable Switch of FUS Phase Separation

Jenny L Carey[1, †], Miyuki Hayashi[1, †], Emily Welebob[2], Laura R Ganser[3], Huan Wang[4], Kerry Buckhaults[5], Jacquelyn A. DePierro[1], Zheng Shi[4], James Shorter[6], Sua Myong[3, 7], Aaron Haeusler[2, *], Lin Guo[1, *]

[1]Department of Biochemistry and Molecular Biology, Thomas Jefferson University, Philadelphia, PA, 19107, USA

[2]Jefferson Weinberg ALS Center, Vickie and Jack Farber Institute for Neuroscience, Department of Neuroscience, Thomas Jefferson University, Philadelphia, PA, 19107, USA

[3]Department of Biophysics, Johns Hopkins University, Baltimore, MD 21218. U.S.A.

[4]Department of Chemistry and Chemical Biology, Rutgers University, Piscataway, NJ 08854, USA

[5]Vickie and Jack Farber Institute for Neuroscience, Department of Neuroscience, Thomas Jefferson University, Philadelphia, PA, 19107, USA

[6]Department of Biochemistry and Biophysics, University of Pennsylvania, Philadelphia, PA, 19104, USA

[7]Boston Children's Hospital, Program in Cellular and Molecular Medicine, Boston, MA 02115, USA

† J.L.C and M.H. contributed equally to this work.

* To whom correspondence should be addressed. Email: Lin.Guo@jefferson.edu

Correspondence may also be addressed to Aaron Haeusler: Email: Aaron.Haeusler@jefferson.edu

SUPPLEMENTARY FIGURES and LEGENDS

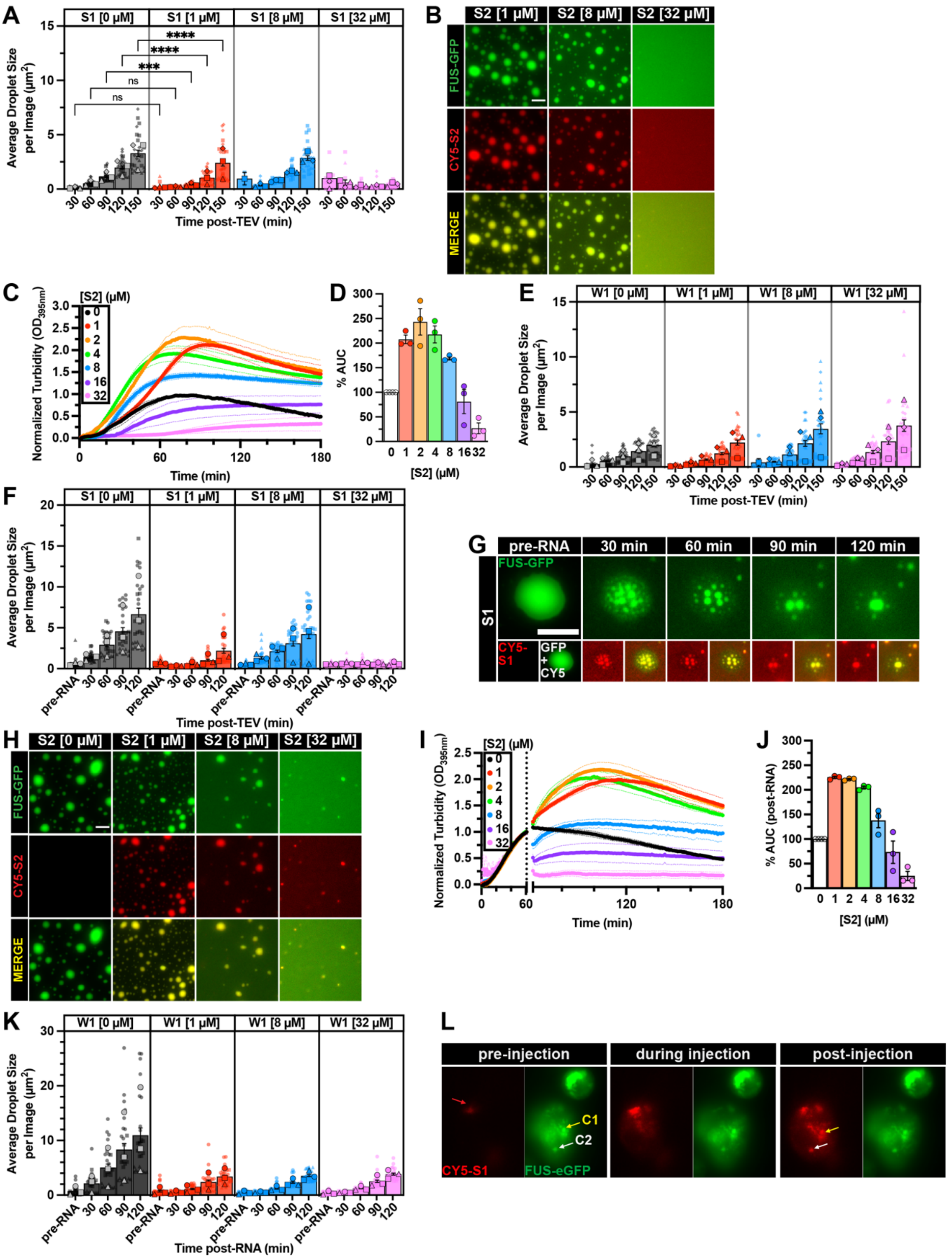

Supplementary Figure 1. Strong RNA inhibitors prevent and reverse FUS phase separation in a concentration-dependent manner.

(**A,E,F,K**) Average droplet size was quantified from images collected in Figures 1B, D (**A**) or Figure 1G (**E**), or from reversal images collected in Fig. 1K (**F**), or Fig. 1O (**K**). Individual points represent image means (n=30), large symbols represent trial means (n=3), and bars represent the mean±SEM (n=30). For Fig. S1A, ordinary one-way ANOVA with Šidák's multiple comparisons test was used to

compare the means between 0 μM S1 and 1 μM S1 droplets at each time point (ns $P>0.05$, ***$P\leq0.0005$, ****$P<0.0001$).

(**B**) Representative images of FUS droplets formed in the presence of RNA S2. LLPS of FUS (1.8 μM FUS supplemented with 0.2 μM FUS-GFP) with S2 (1, 8, or 32 μM unlabeled S2 supplemented with 75 nM Cy5-S2) was monitored by droplet imaging every 30 minutes for a duration of 150 minutes. The 120-minute timepoint images were selected to represent RNA effect on FUS LLPS. Scale bar is 5 μm.

(**C**) Turbidity measurement of FUS LLPS inhibition by RNA S2. MBP-FUS (2 μM) was incubated in the presence of varying concentrations of S2 prior to initiating FUS LLPS reaction with TEV protease. Turbidity at 395 nm was monitored at 25°C over 180 min. Data represent mean±SEM (n=3-4). The 0 μM RNA control plotted in Fig. 1E, 1I, and S1C are the same because these experiments were conducted simultaneously.

(**D**) Area under the curve (AUC) was calculated from turbidity curves in Fig. S1C. Data represent mean±SEM (n=3-4).

(**G**) Zoomed representative images of the same FUS droplets before and after the addition of RNA S1 to illustrate strong RNA "breaking apart" a large and highly-enriched FUS droplet into numerous smaller droplets. FUS (1.8 μM FUS supplemented with 0.2 μM FUS-GFP) droplets were formed for 60 minutes prior to adding S1 (32 μM unlabeled S1 supplemented with 75 nM Cy5-S1). Scale bar is 5 μm.

(**H**) Representative images of FUS droplet 60 minutes after addition of RNA S2. FUS (1.8 μM FUS supplemented with 0.2 μM FUS-GFP) droplets were formed for 60 minutes prior to adding S2 (1, 8, or 32 μM unlabeled S2 supplemented with 75 nM Cy5-S2). Droplets were monitored by imaging immediately before RNA, then every 30 minutes for a duration of 120 minutes after RNA was added. The 60-minute post-RNA timepoint images were selected to represent RNA's effect on FUS LLPS. Scale bar is 5 μm.

(**I**) Turbidity measurement of FUS LLPS reversal with RNA S2. FUS (2 μM) LLPS was initiated, and monitored by turbidity at 395 nm. After 60 min, data collection was paused to add S2, then resumed to monitor turbidity changes for an additional 120 min. Data represent mean±SEM (n=3-4).

(**J**) AUC was calculated from turbidity curves in Fig. S1I after RNA was added. Data represent mean±SEM (n=3-4).

(**L**) Microinjection of Cy5-RNA S1 (0.5 μM) into HEK 293T cells expressing FUS-WT-eGFP. The red arrow marks the site of RNA injection. White and yellow arrows mark Cy5-S1 localization to cytoplasmic FUS condensates ("C1" or "C2") post-injection.

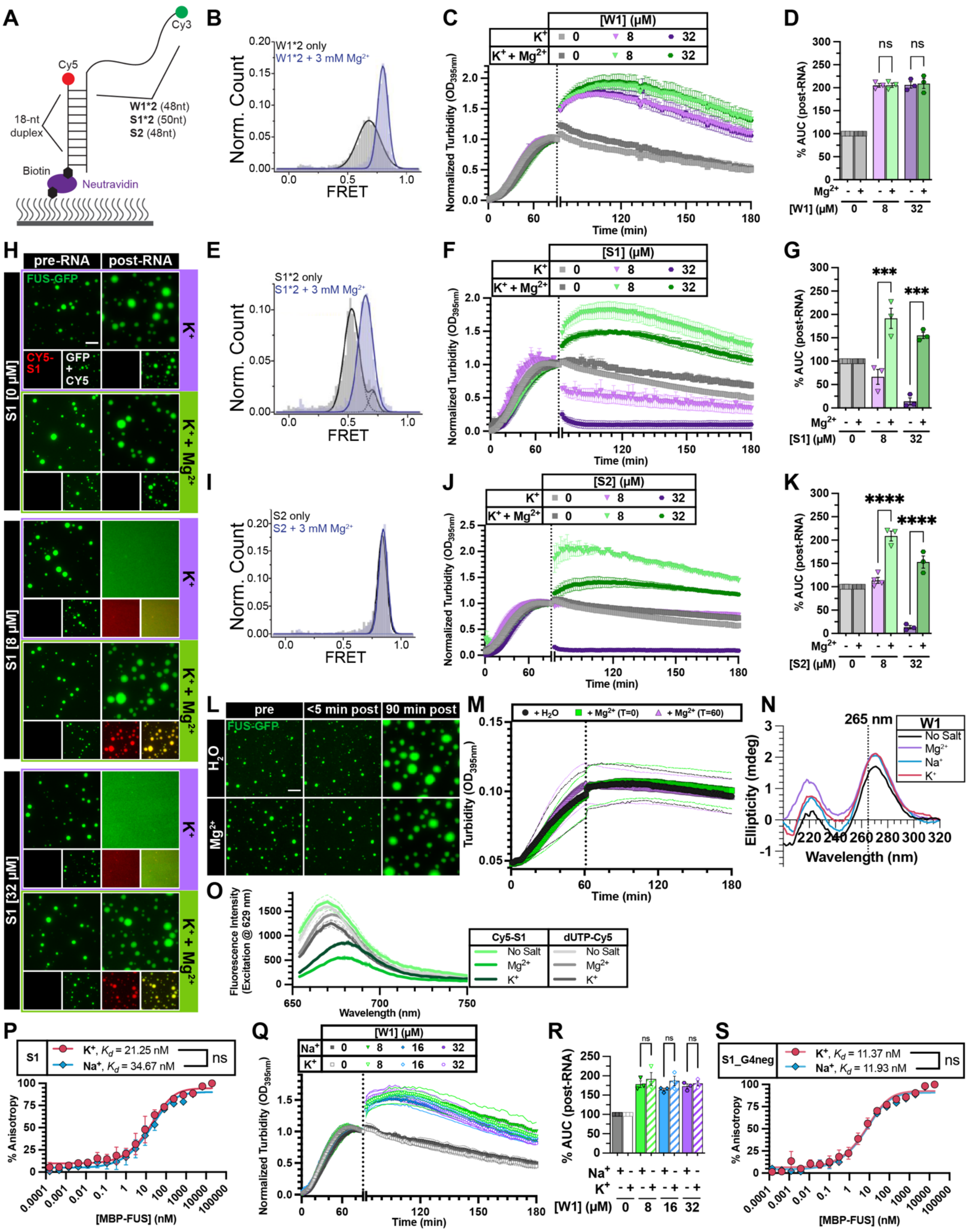

Supplementary Figure 2. rG4 is the specific structural determinant for RNA S1 activity in mitigating FUS LLPS.

(**A**) smFRET experimental diagram. RNA W1 (24nt) and S1 (25nt) were doubled (W1*2 or S1*2) to achieve optimal length for smFRET experiments.

(**B, E, I**) FRET efficiency histogram determined by smFRET experiment for RNA W1*2 (**B**), RNA S1*2 (**E**), or RNA S2 (**I**) in the presence of 100 mM KCl (black trace) or 100 mM KCl supplemented with 3 mM $MgCl_2$ (blue trace).

(**C, F, J**) Turbidity measurement of FUS LLPS reversal with RNA W1 (**C**), S1 (**F**), or S2 (**J**) in TAB-K^+ ± Mg^{2+}. FUS (2 µM) LLPS was initiated, and monitored by turbidity at 395 nm. After 90 min, data collection was paused to add RNA diluted in 10 mM Tris-HCl pH 7.4 supplemented with 50 mM KCl (purple symbols) or 50 mM KCl + 3 mM $MgCl_2$ (green symbols). Data represent mean±SEM (n=3-6). The 0 µM K^+ control traces are the same for the following because these experiments were conducted simultaneously: Figs. 2E & S2F or Figs. S2J & S3C.

(**D, G, K**) AUC was calculated from turbidity curves in Fig. S2C (**D**), Fig. S2F (**G**), or Fig. S2J (**K**) after RNA was added. Data represent mean±SEM (n=3-6). Ordinary one-way ANOVA with Šidák's multiple comparisons test was used to compare the means (ns P>0.05, ***P≤0.0005, ****P<0.0001).

(**H**) Representative images of FUS droplets in TAB-K^+ ± 3 mM $MgCl_2$ before and after the addition of RNA S1. FUS (1.8 µM FUS supplemented with 0.2 µM FUS-GFP) droplets were formed in TAB-K^+ ± 3 mM $MgCl_2$ for 90 minutes prior to adding S1 (8 or 32 µM unlabeled RNA supplemented with 75 nM Cy5-RNA). Images were acquired before adding RNA ("pre-RNA") and 60 min post-RNA addition ("post-RNA"). Scale bar is 5 µm.

(**L**) Representative images of FUS droplets in TAB-K^+ before and after the addition of water (top) or Mg^{2+} (bottom). FUS (1.8 µM FUS supplemented with 0.2 µM FUS-GFP) droplets were formed in TAB-K^+ for 90 minutes prior to adding water or 3 mM Mg^{2+}. Images were acquired before ("pre") and immediately ("<5 min post") or 90 minutes ("90 min post") after adding water or Mg^{2+}. Scale bar is 5 µm.

(**M**) Raw turbidity measurements of FUS LLPS in TAB-K^+ with Mg^{2+} added before ("T=0"; green) or 60 minutes after ("T=60"; purple trace) TEV was added. FUS (2 µM) LLPS was initiated by addition of TEV and monitored by turbidity at 395 nm. Data represent mean±SEM (n=4).

(**N**) Representative CD spectra of RNA W1 collected at 25°C in indicated buffer condition.
(**O**) Emission spectrum of Cy5-S1 or dUTP-Cy5 in No Salt, K^+, or Mg^{2+}. Cy5-S1 (20 nM; green traces) or dUTP-Cy5 (20 nM; gray traces) were excited at 629 nm and fluorescence spectrum was collected from 654 nm - 750 nm to observe emission peak. Data plotted represent mean±SEM (n=3).

(**P, S**) Anisotropy measurement of fluorescein (fl)-RNA S1 (**P**) or fluorescein-RNA S1_G4neg (**S**) with MBP-FUS. For each trial, fl-RNA (8 nM) was folded then added to increasing concentrations of MBP-FUS in TAB-Na^+ (blue diamonds) or TAB-K^+ (red circles). Fluorescence anisotropy was normalized, and the mean±SEM of three independent trials was plotted. Solid lines represent the fitted curve to obtain K_d. Extra sum-of-squares F Test was used to compare K_d in TAB-Na^+ and TAB-K^+ (ns P>0.05).

(**Q**) Turbidity measurement of FUS LLPS reversal with W1 in TAB-Na^+ (closed symbols) or TAB-K^+ (open symbols). LLPS of FUS (2 μM) was initiated, and monitored by turbidity at 395 nm. After 90 min, data collection was paused to add W1. Increasing concentration of RNA is indicated by a more saturated color. Data represent mean±SEM (n=3).

(**R**) AUC was calculated from turbidity curves in Fig. S2Q after RNA was added. Data represent mean±SEM (n=3). Ordinary one-way ANOVA with Šidák's multiple comparisons test was used to compare the means between TAB-Na^+ and TAB-K^+ at each RNA concentration (ns P>0.05).

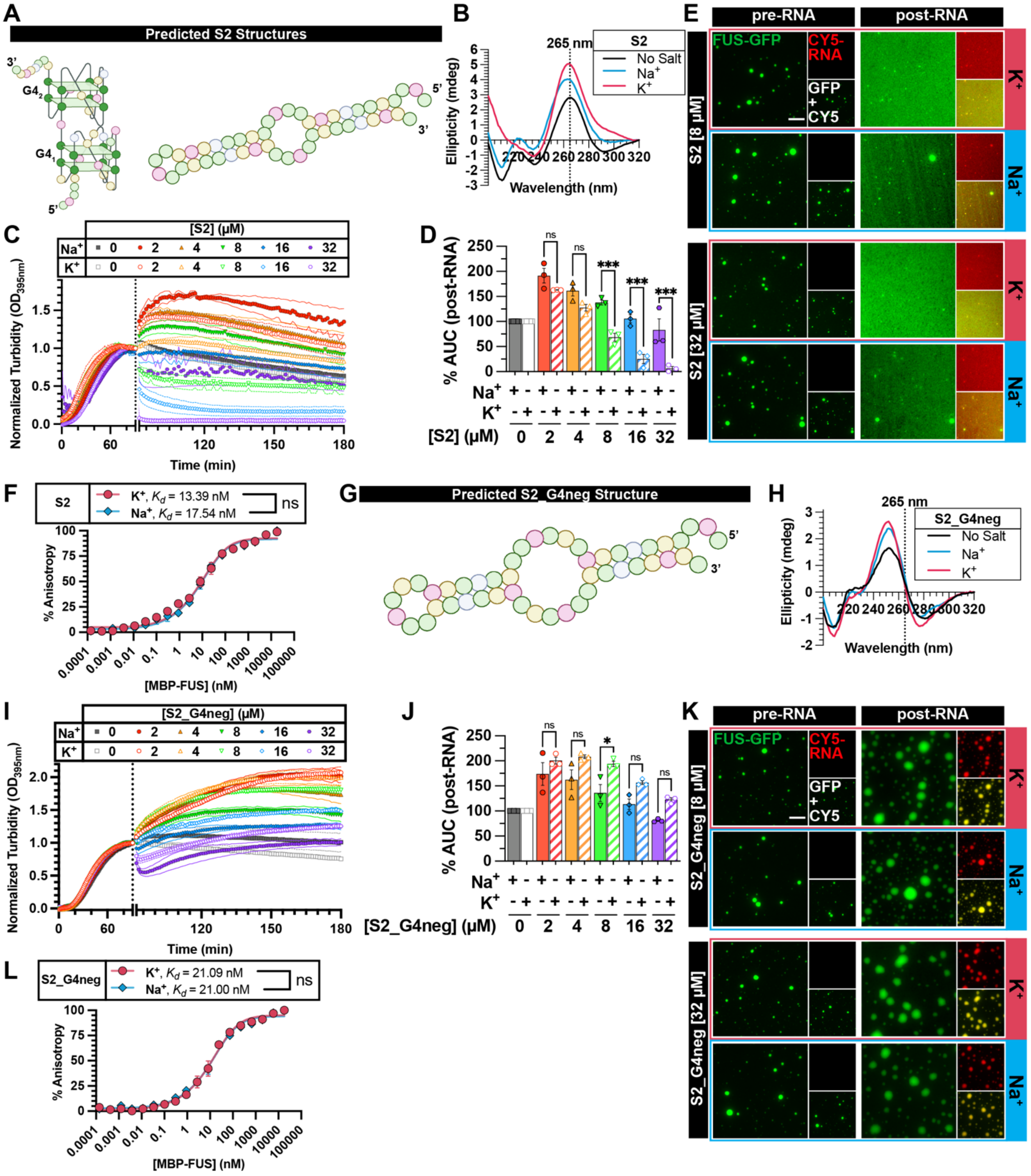

Supplementary Figure 3. rG4, not hairpin, structure also determines RNA S2 activity in mitigating FUS LLPS.

(**A, G**) Schematic representations of predicted rG4 and/or stem-loop structures formed by RNA S2 (**A**) or S2_G4neg (**G**). The rG4 structure was predicted using QGRS Mapper [1] , and the canonical secondary structure was predicted using RNAfold WebServer [2]. RNA structures were created with Biorender.com.

(**B, H**) CD spectra of RNA S2 (**B**) or S2_G4neg (**H**) collected at 25°C in indicated buffer condition.

(**C, I**) Turbidity measurement of FUS LLPS reversal with RNA S2 (**C**) or S2_G4neg (**I**) in TAB-Na^+ (closed symbols) or TAB-K^+ (open symbols). LLPS of FUS (2 μM) was initiated, and monitored by turbidity at 395 nm. After 90 min, data collection was paused to add RNA. Increasing concentration

of RNA is indicated by a more saturated color. Data represent mean±SEM (n=3). The 0 µM RNA control in TAB-K^+ plotted in Fig. S3C and Fig. S2J are the same because these experiments were conducted simultaneously.

(**D, J**) AUC was calculated from turbidity curves in Fig. S3C (**D**) or Fig. S3I (**J**) after RNA was added. Data represent mean±SEM (n=3). Ordinary one-way ANOVA with Šidák's multiple comparisons test was used to compare the means between TAB-Na^+ and TAB-K^+ at each RNA concentration (ns P>0.05, *P≤0.05, ***P≤0.001).

(**E, K**) Representative images of FUS droplets before and after the addition of RNA S2 (**E**) or S2_G4neg (**K**). LLPS of FUS (1.8 µM FUS supplemented with 0.2 µM FUS-GFP) was monitored by droplet imaging before and 60 minutes after the addition of RNA (8 or 32 µM unlabeled RNA supplemented with 75 nM Cy5-RNA). Scale bar is 5 µm.

(**F, L**) Anisotropy measurement of fluorescein-RNA S2 (**F**) or fluorescein-RNA S2_G4neg (**L**) with MBP-FUS. For each trial, fl-RNA (8 nM) was folded then added to increasing concentrations of MBP-FUS in TAB-Na^+ (blue diamonds) or TAB-K^+ (red circles). Fluorescence anisotropy was normalized, and the mean±SEM of three independent trials was plotted. Solid lines represent the fitted curve to obtain K_d. Extra sum-of-squares F Test was used to compare K_d in TAB-Na^+ and TAB-K^+ (ns P>0.05).

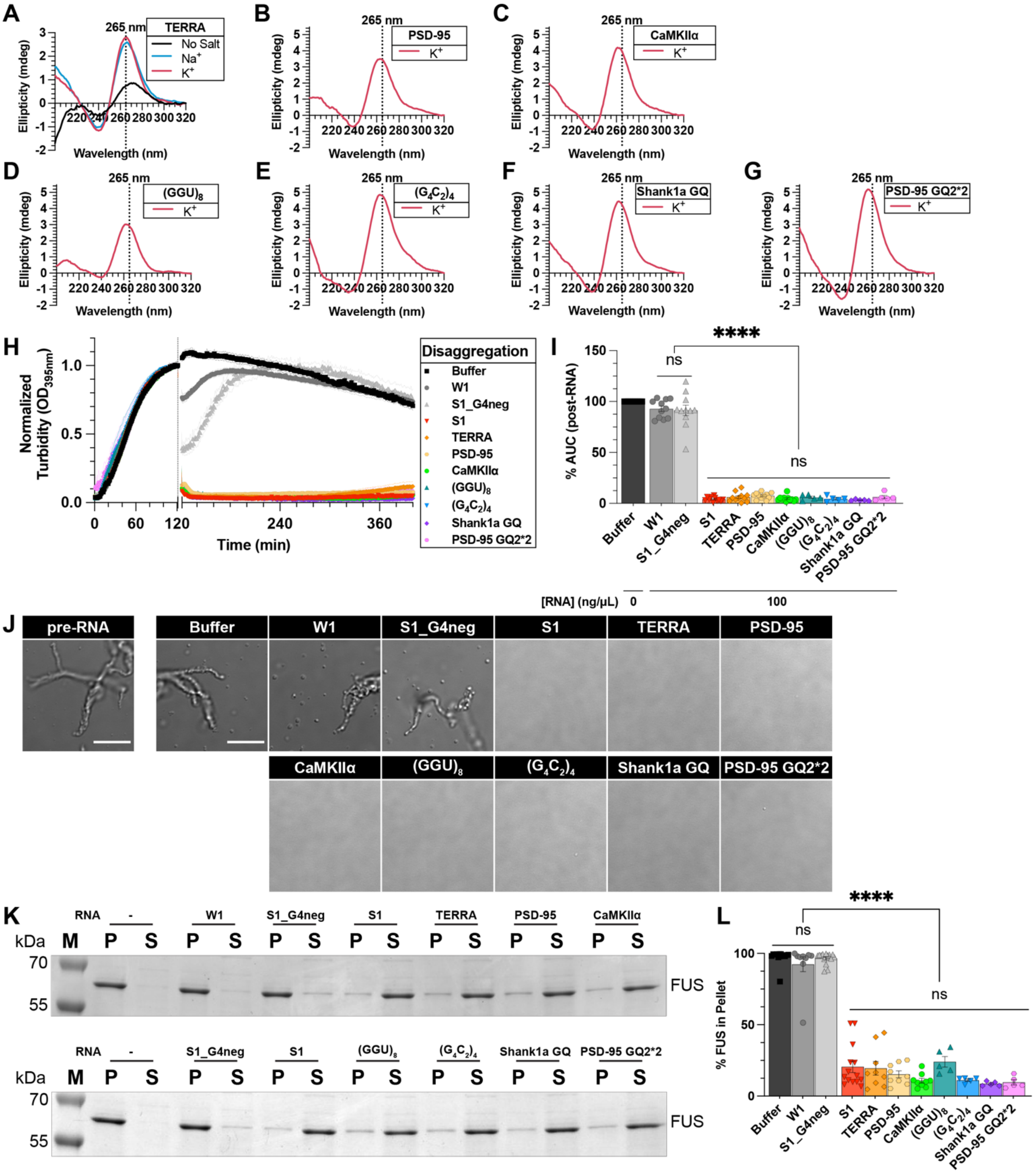

Supplementary Figure 4. FUS-binding rG4 is a defining criterion for identifying strong RNA inhibitors of FUS LLPS and aggregation.

(**A-G**) Representative CD spectra of RNA TERRA (**A**), PSD-95 (**B**), CaMKIIα (**C**), $(GGU)_8$ (**D**), $(G_4C_2)_4$ (**E**), Shank1a GQ (**F**), or PSD-95 GQ2*2 (**G**) collected at 25°C.

(**H**) Turbidity measurement of FUS disaggregation with indicated rG4 RNAs and non-rG4 RNA controls. FUS (5 μM) aggregation was initiated by adding TEV protease in GST-TEV-FUS in Assembly Buffer and monitored by turbidity at 395 nm. After 120 min, data collection was paused to add RNA (100 ng/μL). Data represent mean±SEM (n=5-11).

(**I**) AUC was calculated from turbidity curves in Fig. S4H after RNA was added. Data represent mean±SEM (n=5-11). Ordinary one-way ANOVA with Tukey's multiple comparisons test was used to compare the means between the RNA treatments. (ns P>0.05, ****P<0.0001).

(**J**) Representative differential interference contrast (DIC) images of FUS aggregates before and after the addition of RNA. Preformed FUS aggregates were assembled as in Fig. S4H and images were acquired before ("pre-RNA") or 90 min after addition of buffer or RNA (100 ng/μL). Scale bar is 10 μm.

(**K**) Representative sedimentation analysis gels. FUS (5 μM) aggregation was initiated as in Fig. S4H. After 120 min, RNAs (100 ng/μL) were added and incubated at room temperature. Samples were collected 120 min after the addition of buffer or RNA and analyzed by sedimentation followed by SDS-PAGE.

(**L**) Quantification of SDS-PAGE gel images from Fig. S4K. Data represent mean±SEM (n=5-9). Ordinary one-way ANOVA with Tukey's multiple comparisons test was used to compare the means between the conditions. (ns P>0.05, ****P<0.0001).

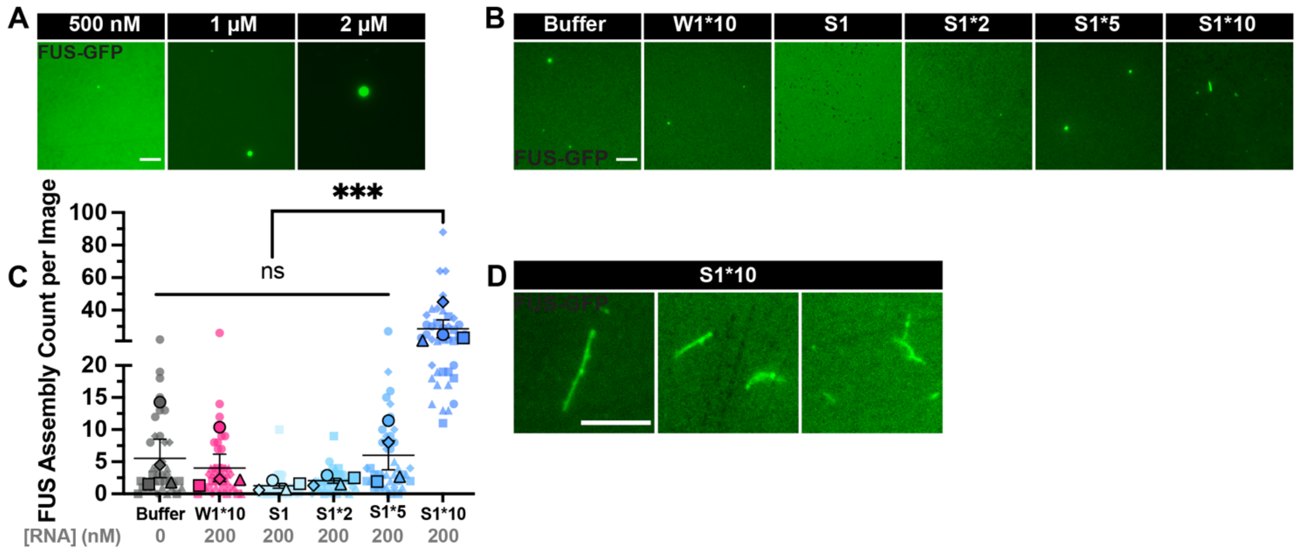

Supplementary Figure 5. Increasing rG4 repeat numbers in RNA S1 switches it from inhibiting to nucleating FUS assembly.

(**A**) Representative images of FUS droplets formed by varying concentrations of FUS-GFP in TAB-K^+. LLPS of FUS-GFP (500 nM, 1 µM, or 2 µM) in TAB-K^+ was initiated by adding TEV protease to cleave MBP tag from MBP-TEV-FUS-GFP. Images were acquired 120 minutes after TEV addition. Scale bar is 5 µm.

(**B**) Representative images of FUS assembly formed with 200 nM of W1*10, S1, S1*2, S1*5, or S1*10 in TAB-K^+. LLPS of FUS-GFP (500 nM) in TAB-K^+ was initiated as in (A) in the presence of indicated RNAs. For each trial, images were acquired 120 minutes after TEV addition (n=4). Scale bar is 5 µm.

(**C**) The average number of FUS assemblies per image was quantified from images collected in Fig. S5B and Fig. 4J. Bars represent mean±SEM of four independent experiments, large symbols represent trial means (n=4), and small datapoints represent individual image values (n=40). The 0 µM RNA control, W1*10 (200 nM), and S1*10 (200 nM) data plotted in Fig. S5C and Fig. 4K are the same because these experiments were conducted simultaneously. Ordinary one-way ANOVA with Tukey's multiple comparisons test was used to compare the means between RNAs. (ns $P>0.05$, ***$P\leq0.0005$).

(**D**) Representative images of FUS assemblies formed in the presence of S1*10. LLPS of FUS-GFP (500 nM) in TAB-K^+ was initiated as in (A) in the presence of S1*10 (200 nM). Images were acquired 120 minutes after TEV addition. Scale bar is 5 µm.

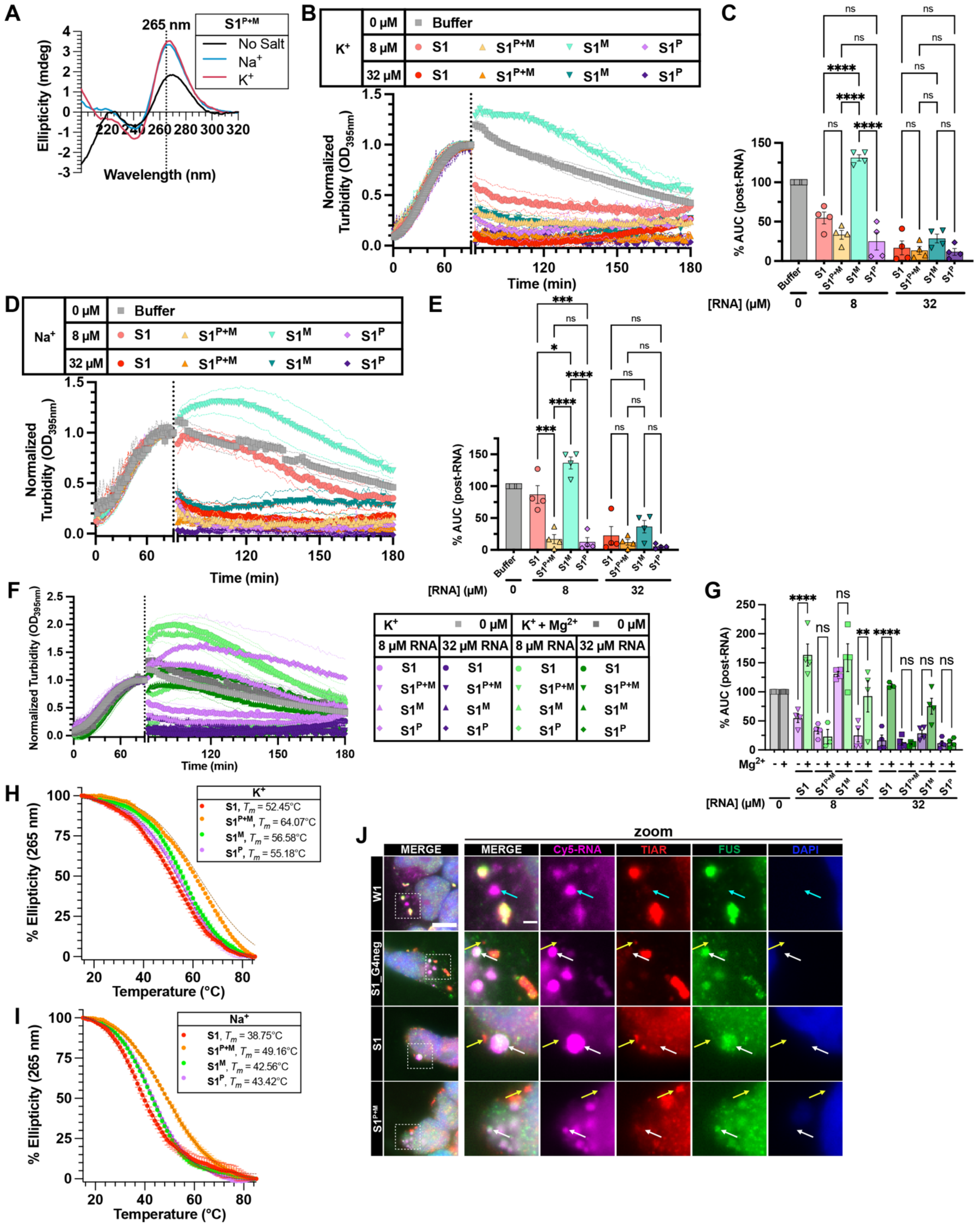

Supplementary Figure 6. Chemical modifications enhancing rG4 stability strengthen RNA activity against FUS phase separation.

(**A**) Representative CD spectra of RNA $S1^{P+M}$ collected at 25°C in indicated buffer.

(**B, D**) Turbidity measurement of FUS LLPS reversal with indicated RNA in TAB-K^+ (**B**) or TAB-Na^+ (**D**). LLPS of FUS (2 μM) was initiated, and monitored by turbidity at 395 nm. After 90 min, data collection was paused to add RNA. Data represent mean±SEM (n=4).

(**C, E**) AUC was calculated from turbidity curves in Fig. S6B (**C**) or Fig. S6D (**E**) after RNA was added. Data represent mean±SEM (n=4). Ordinary one-way ANOVA with Šidák's multiple comparisons test was used to compare the means within each concentration (ns P>0.05, *P≤0.05, ***P≤0.0005, ****P<0.0001).

(**F**) Turbidity measurement of FUS LLPS reversal with indicated RNA in TAB-K^+ ± Mg^{2+}. FUS (2 μM) LLPS was initiated, and monitored by turbidity at 395 nm. After 90 min, data collection was paused to add RNA diluted in 10 mM Tris-HCl pH 7.4 supplemented with 50 mM KCl (purple symbols) or 50 mM KCl + 3 mM $MgCl_2$ (green symbols). Data represent mean±SEM (n=3-4).

(**G**) AUC was calculated from turbidity curves in Fig. S6F after RNA was added. Data represent mean±SEM (n=3-4). Ordinary one-way ANOVA with Šidák's multiple comparisons test was used to compare the means (ns P>0.05, **P≤0.005, ****P<0.0001).

(**H, I**) Thermal melt curves of RNA S1 (red), $S1^{P+M}$ (orange), $S1^{M}$ (green), or $S1^{P}$ (purple) in K^+ (**H**) or Na^+ (**I**) collected at 265 nm. Data represent mean±SEM (n=3). Dashed lines represent the fitted curves used to obtain T_m.

(**J**) Representative images of permeabilized HEK 293 cells treated with 1 μM Cy5-labeled RNA W1, S1_G4neg, S1 or $S1^{P+M}$ after sodium arsenite treatment (0.5 mM, 1 h). Cells were fixed and stained for endogenous FUS (green), TIAR (red), and DAPI (blue). White arrows point to Cy5-RNA colocalized with FUS-positive and TIAR-negative foci. Yellow arrows point to TIAR foci devoid of FUS or Cy5-RNA. Blue arrows point to Cy5-RNA foci outside of FUS or TIAR foci. Merged scale bar is 10 μm. Zoom scale bar is 2 μm.

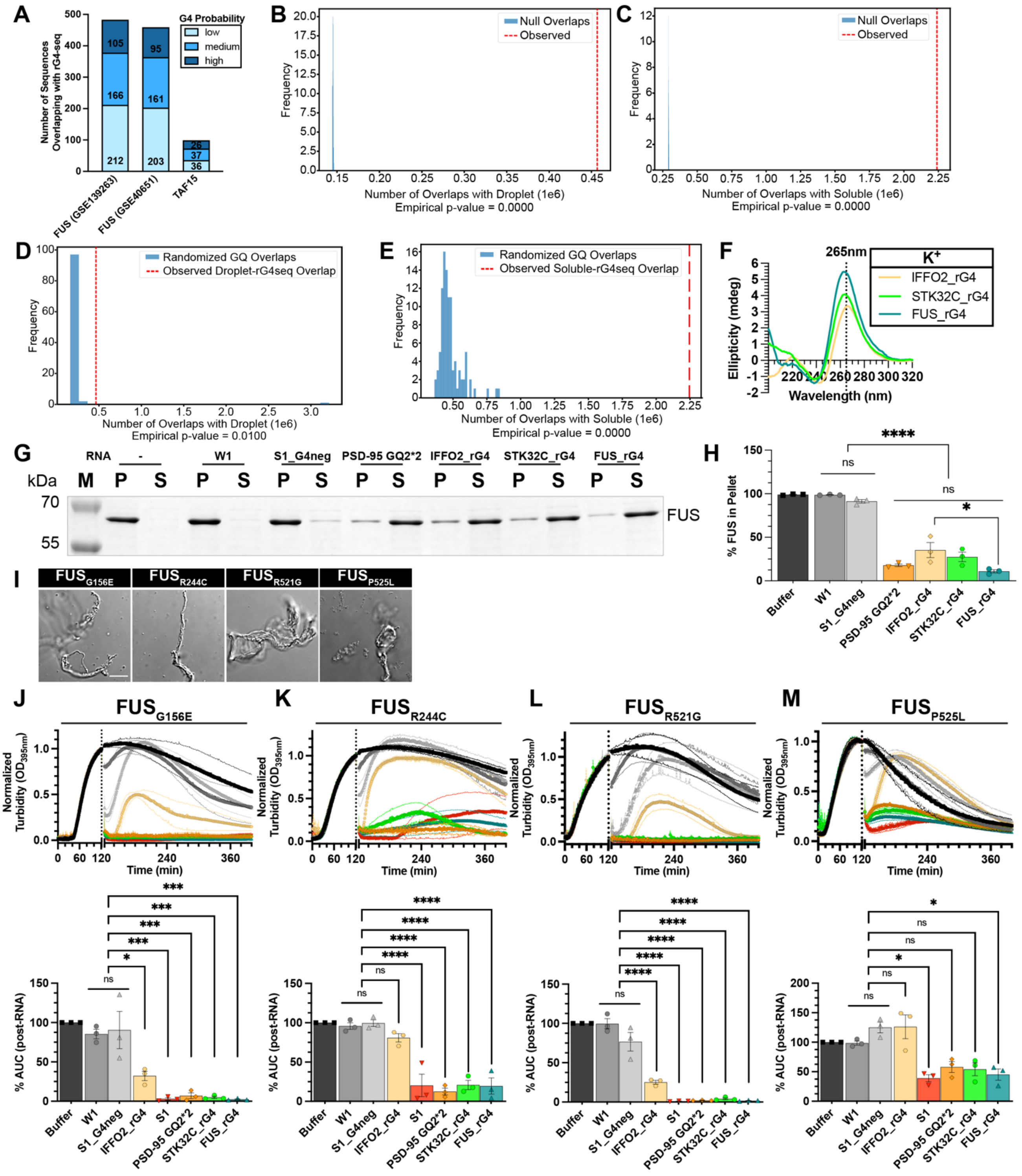

Supplementary Figure 7. Transcriptome-wide bioinformatic analysis identifies novel RNA inhibitors of FUS LLPS and aggregation.

(**A**) Number of sequences shared between FUS CLIP [3, 4] or TAF15 CLIP [5] and rG4 database [6]. Overlap sequences with high probability of forming rG4 are in dark blue, those with medium probability are in blue, and those with low probability are in light blue.

(**B-C**) Permutation testing querying whether FUS droplet interactome (**B**) or FUS soluble interactome (**C**) specifically overlap with the rG4 database. rG4 regions were overlapped with randomly-generated regions from hg38 human transcriptome. Each randomly-generated file contained a number of regions consistent with the FUS droplet interactome or FUS soluble interactome files. This testing was

repeated 100 times each for (**B**) and (**C**). Overlaps for each of the 100 tests were recorded (blue) and compared to the observed overlap between FUS droplet or soluble interactomes and rG4 database (red line).

(**D-E**) Permutation testing querying whether the rG4 database specifically overlaps with the FUS droplet interactome (**D**) or FUS soluble interactome (**E**) more than other regions. FUS droplet (**D**) or soluble (**E**) interactomes were tested against randomly-generated 30 nt regions from the human transcriptome. These randomly-generated files were chromosome-matched and consistent with the number of regions originally in the rG4 database. Each test was repeated 100 times for FUS droplet and soluble interactomes. Overlaps for each of the 100 tests were recorded (blue) and compared to the observed overlap between FUS droplet or soluble interactomes and rG4 database (red line).

(**F**) Representative CD spectra of indicated RNAs collected at 25°C.

(**G**) Representative sedimentation analysis gels. FUS (5 μM) aggregation was initiated as in Fig. 6H. After 120 min, RNAs (100 ng/μL) were added and incubated at room temperature. Samples were collected 120 min after the addition of buffer or RNA and analyzed by sedimentation followed by SDS-PAGE.

(**H**) Quantification of SDS-PAGE images from Fig. S7G. FUS bands were quantified in pellet and supernatant fractions, and % FUS in pellet is reported. Data represent mean±SEM (n=3). Ordinary one-way ANOVA with Tukey's multiple comparisons test was used to compare the means between the conditions. (ns P>0.05, *P≤0.05, ****P<0.0001).

(**I**) Representative differential interference contrast (DIC) images of mutant FUS aggregates before addition of RNA. Scale bar is 10 μm.

(**J-M**) Turbidity measurements (top panels) of FUS_{G156E} (**J**), FUS_{R244C} (**K**), FUS_{R521G} (**L**), or FUS_{P525L} (**M**) disaggregation with indicated rG4 RNAs and non-rG4 RNA controls. FUS (5 μM) aggregation was initiated by adding TEV protease to GST-TEV-FUS in Assembly Buffer and monitored by turbidity at 395 nm. After 120 min, data collection was paused to add RNA (100 ng/μL). Data represent mean±SEM (n=3). AUC (bottom panels) was calculated from respective turbidity curves after RNA was added. Data represent mean±SEM (n=3). Ordinary one-way ANOVA with Šidák's multiple comparisons test was used to compare the means between the RNA treatments. All comparisons tested are plotted (ns P>0.05, *P≤0.05, ***P≤0.001, ****P<0.0001).

SUPPLEMENTARY TABLES

Supplementary Table 1. RNA oligonucleotides used in study. QGRS Mapper [1] was used to predict whether RNAs would form rG4.

Oligonucleotide	Sequence (5' → 3')<br><br>mX = 2'-O-methylation base<br>X(*) = phosphorothioate linkage	Length	Predicted to Form G4?	Oligonucleotide Reference
S1	CUAGGAUGGAGGUGGGGAAUGG UAC	25	Yes	Guo et al. [7]
S1^{P+M}	mC(*)mU(*)mA(*)mG(*)mG(*)mA(*) mU(*)mG(*)mG(*)mA(*)mG(*)mG(*)mU(*)mG(*)mG(*)mG(*)mG(*)mA(*)mA(*)mU(*)mG(*)mG(*)mU (*)mA(*)mC	25	Not Tested	Guo et al. [7]
S1^{M}	mCmUmAmGmGmAmUmGmGmAm GmGmUmGmGmGmGmAmAmUmG mGmU mAmC	25	Not Tested	Guo et al. [7]
S1^{P}	C(*)U(*)A(*)G(*)G(*)A(*)U(*)G(*)G (*)A(*)G(*)G(*)U(*)G(*)G(*)G(*)G(*)A(*)A(*)U(*)G(*)G(*)U (*)A(*)C	25	Not Tested	This Paper
S1*2	CUAGGAUGGAGGUGGGGAAUGG UACCUAGGAUGGAGGUGGGGAA UGGUAC	50	Yes	This Paper
S1*5	GGCUAGGAUGGAGGUGGGGAAU GGUACCUAGGAUGGAGGUGGGG AAUGGUACCUAGGAUGGAGGUG GGGAAUGGUACCUAGGAUGGAG GUGGGGAAUGGUACCUAGGAUG GAGGUGGGGAAUGGUAC	127	Yes	This Paper
S1*10	GGCUAGGAUGGAGGUGGGGAAU GGUACCUAGGAUGGAGGUGGGG AAUGGUACCUAGGAUGGAGGUG GGGAAUGGUACCUAGGAUGGAG GUGGGGAAUGGUACCUAGGAUG GAGGUGGGGAAUGGUACCUAGG AUGGAGGUGGGGAAUGGUACCU AGGAUGGAGGUGGGGAAUGGUA CCUAGGAUGGAGGUGGGGAAUG GUACCUAGGAUGGAGGUGGGGA AUGGUACCUAGGAUGGAGGUGG GGAAUGGUAC	252	Yes	This Paper
S1_G4neg	GGCGUGAGAGUAGCGAUGUGUA GAG	25	No	This Paper
S2	GAGGUGGCUAUGGAGGUGGCUA UGGAGGUGGCUAUGGAGGUGGC UAUG	48	Yes	Guo et al. [7]
S2_G4neg	GAGGUGCGUAGUGAGGUGCGUA GUGAGGUGCGUAGUGAGGUGCG UAUG	48	No	This Paper
W1	UCAGAGACAUCAUCAGAGACAU CA	24	No	Guo et al. [7]

W1*2	UCAGAGACAUCAUCAGAGACAU<br>CAUCAGAGACAUCAUCAGAGAC<br>AUCA	48	No	This Paper
W1*10	GGUCAGAGACAUCAUCAGAGAC<br>AUCAUCAGAGACAUCAUCAGAG<br>ACAUCAUCAGAGACAUCAUCAG<br>AGACAUCAUCAGAGACAUCAUC<br>AGAGACAUCAUCAGAGACAUCA<br>UCAGAGACAUCAUCAGAGACAU<br>CAUCAGAGACAUCAUCAGAGAC<br>AUCAUCAGAGACAUCAUCAGAG<br>ACAUCAUCAGAGACAUCAUCAG<br>AGACAUCAUCAGAGACAUCAUC<br>AGAGACAUCAUCAGAGACAUCA	242	No	This Paper
TERRA	UUAGGGUUAGGGUUAGGGUUAG<br>GG	24	Yes	Wang et al. [8]
$(GGU)_8$	GGUGGUGGUGGUGGUGGUGGUG<br>GU	24	Yes	This Paper
$(G_4C_2)_4$	GGGGCCGGGGCCGGGGCCGGGG<br>CC	24	Yes	Fujino et al. [9]
PSD-95	GGGGAAAAGGGAGGGAUGGG	20	Yes	Ishiguro et al. [10]
CaMKIIα	UGGGGGGGGCGGGUGGGAUGGG<br>A	23	Yes	Ishiguro et al. [10]
Shank1a GQ	GGGGUUGGGGAGGGUGUAGGGG<br>GUGGGG	28	Yes	Imperatore et al. [11]
PSD-95 GQ2*2	GGGAGGGAGGGUGGGGGGAGGG<br>AGGGUGGG	30	Yes	Imperatore et al. [11]<br>*Doubled length of oligo
IFFO2_rG4	GCAGGAAAUUCAGAAAUGUGGU<br>GGGUGGGG	30	Yes	Kwok et al. [6]
STK32C_rG4	GUCAAGGGCAUGGGUUGGGGUA<br>GUGGGUGG	30	Yes	Kwok et al. [6]
FUS_rG4	GGAGGCUUCCGAGGGGGCCGGG<br>GUGGUGGG	30	Yes	Kwok et al. [6]

Supplementary Table 2. QGRS Mapper Results of previously-identified RNA inhibitors. QGRS Mapper [1] was used to predict whether RNAs would form rG4. "S" denotes a strong inhibitor, "W" denotes a weak inhibitor, and "N" denotes no activity. If RNA is predicted to form rG4, major position(s), length(s), and G-Score(s) are reported.

Oligonucleotide	Sequence (5' → 3')<br>Predicted rG4 sequence is underlined	Predicted to form rG4?	rG4 Position	rG4 Length	rG4 G-Score
S1	CUAGGAUGGAGGUGGGGAAUGGUAC	YES	4	13	20
S2	GAGGUGGCUAUGGAGGUGGCUAUGGAGGUGGCUAUGGAGGUGGCUAUG	YES	6	20	21
			27	14	18
S3	AUUGAGGAGCAGCAGAGAAGUUGGAGUGAAGGCAGAGAGGGGUUAAGG	YES	23	26	21
S4	ACCAUGAUCACGAAGGUGGUUUUCCCAGGGCGAGGCUUA	YES	15	21	14
S5	CUCCGGAUGUGCUGACCCCUGCGAUUUCCCCAAAUGUGGGAAA	NO			
S6	AAAAAAAAAAAAAAAAAAAAAAAAAGUGAAGGCAGAGAGGGGUUAAGG	YES	31	18	15
S7	GACUGAAAAAGGUGGGUUUCUUUU	NO			
S8	AUUGAGGAGCAGCAGAGAAGUUGGAAAAAAAAAAAAAAAAAAAAAAAAA	NO			
W1	UCAGAGACAUCAUCAGAGACAUCA	NO			
W2	GAAAAUUAAUGUGUGUGUGUGGAAAAUU	NO			
W3	UUGUAUUUUGAGCUAGUUUGGUGAU	NO			
W4	GGUGAGCACAGAGGUGAGCACAGA	NO			
W5	CCAAUCUUCCUUCCAAUCUUCCUU	NO			
W6	GAUGGAUUCCAGGAUGGAUUCCAG	NO			
W7	AAACGGUCUGAUAAACGGUCUGAU	NO			
W8	AAAGCGGCGAUGAAAGCGGCGAUG	NO			
W9	UAUUGAUCCGGUUAUUGAUCCGGU	NO			
N1	CGCUGGCAUCCACGCUGGCAUCCA	NO			
N2	ACAGUCCCCCGGACAGUCCCCCGG	NO			

N3	ACCGGCGAACCGGCGAACCGG CGA	NO			
N4	CAGUAUUAUUUCAGUAUUAU UUU	NO			
N5	CCAUCCAGUCUACCAUCCAGU CUA	NO			
N6	CCAGUCUGGCCCCCAGUCUGG CCC	NO			

REFERENCES

1. Kikin, O., D'Antonio, L. and Bagga, P.S. (2006) QGRS Mapper: a web-based server for predicting G-quadruplexes in nucleotide sequences. *Nucleic Acids Res*, **34**, W676-682.
2. Gruber, A.R., Lorenz, R., Bernhart, S.H., Neubock, R. *et al.* (2008) The Vienna RNA websuite. *Nucleic Acids Res*, **36**, W70-74.
3. Lagier-Tourenne, C., Polymenidou, M., Hutt, K.R., Vu, A.Q. *et al.* (2012) Divergent roles of ALS-linked proteins FUS/TLS and TDP-43 intersect in processing long pre-mRNAs. *Nat Neurosci*, **15**, 1488-1497.
4. Jutzi, D., Campagne, S., Schmidt, R., Reber, S. *et al.* (2020) Aberrant interaction of FUS with the U1 snRNA provides a molecular mechanism of FUS induced amyotrophic lateral sclerosis. *Nat Commun*, **11**, 6341.
5. Ibrahim, F., Maragkakis, M., Alexiou, P., Maronski, M.A. *et al.* (2013) Identification of in vivo, conserved, TAF15 RNA binding sites reveals the impact of TAF15 on the neuronal transcriptome. *Cell Rep*, **3**, 301-308.
6. Kwok, C.K., Marsico, G., Sahakyan, A.B., Chambers, V.S. *et al.* (2016) rG4-seq reveals widespread formation of G-quadruplex structures in the human transcriptome. *Nat Methods*, **13**, 841-844.
7. Guo, L., Mann, J.R., Mauna, J.C., Copley, K.E. *et al.* (2026) Defining RNA oligonucleotides that reverse deleterious phase transitions of RNA-binding proteins with prion-like domains. *Molecular Cell*, **86**, 114-134.e110.
8. Wang, X., Schwartz, J.C. and Cech, T.R. (2015) Nucleic acid-binding specificity of human FUS protein. *Nucleic Acids Res*, **43**, 7535-7543.
9. Fujino, Y., Ueyama, M., Ishiguro, T., Ozawa, D. *et al.* (2023) FUS regulates RAN translation through modulating the G-quadruplex structure of GGGGCC repeat RNA in C9orf72-linked ALS/FTD. *Elife*, **12**.
10. Ishiguro, A., Lu, J., Ozawa, D., Nagai, Y. *et al.* (2021) ALS-linked FUS mutations dysregulate G-quadruplex-dependent liquid-liquid phase separation and liquid-to-solid transition. *J Biol Chem*, **297**, 101284.
11. Imperatore, J.A., McAninch, D.S., Valdez-Sinon, A.N., Bassell, G.J. *et al.* (2020) FUS Recognizes G Quadruplex Structures Within Neuronal mRNAs. *Front Mol Biosci*, **7**, 6.
